# PharmacoNER Tagger: a deep learning-based tool for automatically finding chemicals and drugs in Spanish medical texts

**DOI:** 10.5808/GI.2019.17.2.e15

**Published:** 2019-06-19

**Authors:** Jordi Armengol-Estapé, Felipe Soares, Montserrat Marimon, Martin Krallinger

**Affiliations:** 1 Universitat Politècnica de Catalunya (UPC), 08034 Barcelona, Spain; 2 Barcelona Supercomputing Center (BSC), 08034 Barcelona, Spain; 3 Centro Nacional de Investigaciones Oncológicas (CNIO), 28029 Madrid, Spain

**Keywords:** machine learning, natural language processing, neural networks (computer)

## Abstract

Automatically detecting mentions of pharmaceutical drugs and chemical substances is key for the subsequent extraction of relations of chemicals with other biomedical entities such as genes, proteins, diseases, adverse reactions or symptoms. The identification of drug mentions is also a prior step for complex event types such as drug dosage recognition, duration of medical treatments or drug repurposing. Formally, this task is known as named entity recognition (NER), meaning automatically identifying mentions of predefined entities of interest in running text. In the domain of medical texts, for chemical entity recognition (CER), techniques based on hand-crafted rules and graph-based models can provide adequate performance. In the recent years, the field of natural language processing has mainly pivoted to deep learning and state-of-the-art results for most tasks involving natural language are usually obtained with artificial neural networks. Competitive resources for drug name recognition in English medical texts are already available and heavily used, while for other languages such as Spanish these tools, although clearly needed were missing. In this work, we adapt an existing neural NER system, NeuroNER, to the particular domain of Spanish clinical case texts, and extend the neural network to be able to take into account additional features apart from the plain text. NeuroNER can be considered a competitive baseline system for Spanish drug and CER promoted by the Spanish national plan for the advancement of language technologies (Plan TL).

**Availability:** PharmacoNER Tagger can be accessed at https://github.com/PlanTL-SANIDAD/PharmacoNER.

## Introduction

Chemical compounds and drugs represent a key biomedical entity of common interest for a range of scientific disciplines, including medicine and clinical research, pharmacology as well as basic biomedical research. Due to the growing amount of clinical texts, medical and biomedical literature and medicinal chemistry patents, a systematic approach to recognize such entities in order to semantically enrich these documents and enable further relation extraction task is needed. Text mining and information extraction efforts are gradually being adopted to empower the transformation of unstructured running texts to more structured data representations that can be directly consumed by content analytics and information retrieval infrastructures [1].

Semantically labeling drug mentions in medical documents is a necessary step to allow a range of downstream text mining applications, like drug-resistance, drug dosage recognition, duration of medical treatments, drug repurposing, detection of medication-related allergies, drug-drug interactions or disease-drug relations. Drug names show some inherent characteristics such as the constraints imposed by the International Nonproprietary Names (INN) system by exploiting certain suffixes and prefixes to group them according to certain attributes. For instance, the suffix ‘-caine’ is usually used for local anesthetics. These properties are not only useful for healthcare professionals and pharmacologists to memorize drug names, their attributes and fining them in the clinical literature, they are also constituting valuable attributes for automatically labeling them through language technology tools.

Named entity recognition (NER) [2] is the task of automatically extracting and identifying mentions of entities of interest in running text, typically through their mention offsets or by classifying individual tokens whether they belong to entity mentions of not. Among many other uses, such as entity-aware machine translation, NER can also be used for the automated information extraction and anonymization [3] of medical texts. Early techniques involved the use of hand-crafted rules. The most successful approach consisted in using Conditional Random Fields (CRFs), a graph-based model for modeling sequences that still relied on features defined by humans. In the last years, Natural Language Processing (NLP) techniques have been improved with the use of word embeddings, that is to say, projections of words to vector spaces such that among other findings, the distance can be used as a semantic similarity measure [4]. Deep learning (e.g., artificial neural networks [ANNs] with a high number of hidden layers) has proved to be extremely useful in solving NER problems. In this article, we introduce PharmacoNER tagger, NER software based on NeuroNER [5] adapted to the domain of Spanish medical texts and improved with non-domain-specific features.

### The problem

Medical documents mentioning drugs and chemical compounds offer a relevant source of information regarding the patients’ treatment. For instance, doctors and researchers may be interested in finding health records of patients treated with a particular kind of compound in order to manually analyze them or use them in biomedical NLP pipelines. Nevertheless, the task of manually detecting these entities would be expensive and time-consuming, which prompts for the development of NER systems for automating this task. In the context of chemical compounds, NER is usually referred to as chemical entity recognition (CER).

### Challenges in NER for medical texts and chemical compounds

NER itself has proven to be a challenging task because of the ambiguity and complexity of human languages. In the particular case of CER, additional difficulties arise.

First of all, not all chemical names present distinctive patterns as far as name segments or chemical word morphology are concerned. CER systems are particularly sensitive with regard to both spelling errors and the tokenization strategy of choice since chemical documents usually exhibit hyphenated text segments or variable use of parentheses, brackets, dots, and other punctuation signs. In addition, chemical documents tend to be loaded with acronyms and abbreviations, which are one of the main sources of false positives.

Another characteristic that makes CER difficult is the fact that the detection of mention boundaries is especially cumbersome when long chemicals or modifiers are present. A more in-depth description of difficulties in tagging chemicals can be found at [6].

### Other approaches

In the past, most NER systems were rule-based or leveraged graph-based models such as CRFs, while nowadays the task is usually approached with deep learning techniques. In the case of CER systems, we can highlight some relevant works.

ChemicalTagger [7] parsed the text with a formal grammar and domain-specific regular expressions (regex) and used the parse tree in combination with the Part-of-Speech (POS) tags obtained with an English tagger in order to extract chemical entities. They reported achieving machine-annotator agreements of 88.9% for phrase recognition and 91.9% for phrase-type identification. The test corpus was assembled by compiling 50 paragraphs from the experimental sections of polymer synthesis related papers.

CheNER [8], a tool for the identification of chemical entities and their classes in the biomedical literature, is based on a combination of CRFs, regex and dictionary matching. An F-Score of about 73% was reported, with minor differences depending on the particular experiment. For evaluation, two corpora containing 3,500 documents with approximately 29,500 annotated chemical entities divided into several classes were used.

On the other hand, MetaMap [8], a tool for recognizing Unified Medical Language System (UMLS) concepts in the text, mapped entities to UMLS concepts by the means of rules and a parser.

In CHEMDNER [6], a drug and chemical names extraction competition, the top scoring teams obtained F-scores of 87.39% and 88.2% depending on the particular task, employing CRFs and domain-specific rules. These scores were obtained in the CHEMDNER corpus, a dataset with chemical compounds manually annotated by domain experts.

With regard to drug recognition, a vast survey on approaches and resources was presented in Liu et al.’s study [9], including classical machine learning techniques and hybrid approaches. The authors suggested using deep learning techniques for drug recognition as future work.

A system for drug name recognition and classification in biomedical texts was introduced in Segura-Bedmar et al.’s study [10]. By combining information obtained by the UMLS MetaMap Transfer (MMTx) program and nomenclature rules recommended by the World Health Organization (WHO) INNs Program, a broader coverage than previous approaches based on standalone MMTx was achieved.

For an extensive review of text mining techniques for drugs and chemical compounds, readers can refer to Vazquez et al.’s study [11].

Nevertheless, the aforementioned works used techniques that have some important limitations [1]. Dictionary-based methods have problems dealing with name variability since chemical naming exhibits high variation. Rule construction, apart from requiring domain knowledge and an extensive manual workload, has proven to be difficult to scale to new rules and to transfer to slightly different domains. On the other hand, statistical models such as CRFs and classic machine learning algorithms are not powerful enough for detecting some patterns and require a certain amount of feature engineering. All these previous approaches focused on data in English, despite the fact that there is a considerable amount of textual data and repositories (e.g., MEDES, Scielo, Ibecs, or Cuiden), as well as a large medical end users community interested in tools for processing data in Spanish. Regarding other languages, this work [12] presented a system based on CRFs that was capable of recognizing entities in French biomedical documents.

### Our proposal

Our proposal involves the adaption of a state-of-the-art NER (i.e., NeuroNER) based on deep learning to the particular domain of the Spanish medical texts in order to identify identities as proteins or other components. Remarkably, the system we base our work on has a CRF layer apart from deep learning components. The original system takes tokens as its input, while our proposal involves using additional features in the neural network.

### Key innovations

Our contribution consists in extending an existing neural NER system with additional features that had worked with classic CERs. In particular, POS tags, gazetteer features, and affixes features have been added to the network and were as a pre-preprocessing step. Different experiments have been conducted in order to determine whether these features improved the performance of the neural network. The extension of the neural network is domain and language-agnostic, while the pre-processing is domain-dependent and specific tools for Spanish medical texts have been used. By the neural network itself being language-agnostic we mean that our system could be adapted to other languages by building gazetteer and affix dictionaries and using a POS tagger trained for the required language.

## NeuroNER

NeuroNER, an open-source program for named-entity recognition, achieved state-of-the-art performance by having a neural architecture containing three layers: (1) character-enhanced token-embedding layer, (2) a label prediction layer, and (3) a label sequence optimization layer.

Recurrent neural networks (RNNs) are ANNs such that the computational graphs have cycles, which are used for dealing with sequences [13]. Long short term memory (LSTM) [14] are gated units widely used in RNN implementations. A Bidirectional LSTM (Bi-LSTM) is an LSTM unit such that two inputs layers with opposite directions are present, which allows the network to get information from both the past and future states simultaneously. NeuroNER makes use of Bi-LSTM for the character-enhanced token-embedding layer, while character embeddings are previously passed through their own LSTM.

**Fig. 1.**
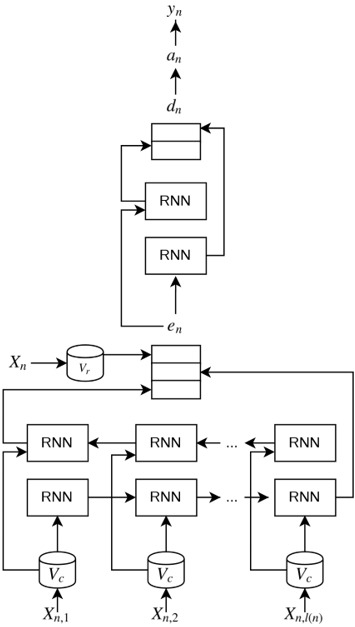
Schematic representation of the artificial neural networks for a single token in NeuroNER: Snippet of the architecture used in NeuroNER. The type of recurrent neural network (RNN) is long short term memory (LSTM). n is the number of tokens *X_i_* is the *i^th^* token. *V_r_* is the mapping from tokens to token embeddings. *l(i)* is the number of characters, and *X_i,j_* is the *j^th^* character of the *i^th^* token. *V_c_* is the mapping from characters to character embeddings. ei is the character-enhanced token embeddings of the *i^th^* token. di is the output of the LSTM of label prediction layer, *a_i_* is the probability vector over labels, and *y_i_* is the predicted label of the *i^th^* token. Adapted from Dernoncourt et al. J Am Med Inform Assoc 2017;24:596-606 [15].

In Fig. 1, we show a representation of the ANN in the original NeuroNER implementation. We must notice that this is just a snippet of the structure of one token. The full ANN has connections for the RNN between tokens from the character-enhanced token embedding upwards. For details about this model, readers can refer to Dernoncourt et al. [15].

In addition, NeuroNER has the ability to load pre-trained token embeddings, which may increase final performance and decrease training time. For more details about NeuroNER, please refer to the following related works: Dernoncourt et al. [5,15].

## Proposed Method

The following features have been added to both the neural network and the dataset parser of NeuroNER:

‒ POS features: with our modification, PharmacoNER tagger can parse annotations (both in BRAT or CONLL formats) of POS tags, encode them with a one-hot encoding for each token and include this in the token embedding layer of the network.

‒ Gazetteer features: our modified version of NeuroNER is able to parse a text file with terms related to the target entities, build a dictionary and assign a positive value for the words that are found in this dictionary. This feature is then concatenated with the character-enhanced token embedding layer. A file with the dictionary has to be supplied by the user.

‒ Affixes features: PharmacoNER tagger can use information regarding affixes in a similar manner as the gazetteer features, but using regular expressions to detect whether a particular word has an affix related to the target entities. A file of affixes shall be supplied by the user.

**Fig. 2.**
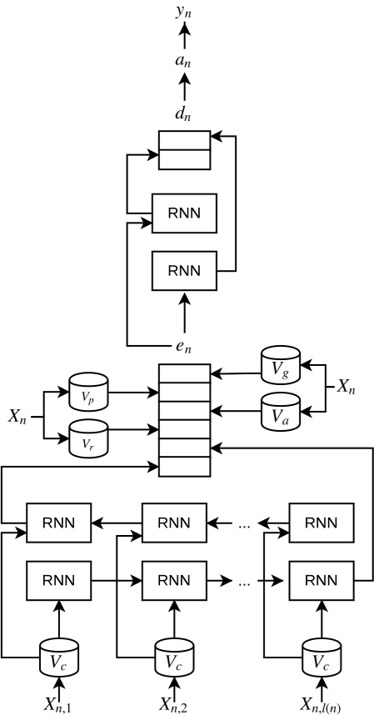
Schematic representation of the the artificial neural networks for a single token in PharmacoNER tagger: Snippet of the architecture used in PharmacoNER tagger. All notations are the same as in Fig. 1. We added the following features in the character-enhanced token embeddings: *V_p_* is the mapping from the specific token to its Part-of-Speech, *V_a_* is boolean mapping to identify if the given token contains any of the affixes in the database, and *V_g_* is a boolean mapping to the gazetteer database. RNN, recurrent neural network.

Notice that the aforementioned features are domain and language independent. The final software can be found in Github (https://github.com/PlanTL-SANIDAD/PharmacoNER), with information about installation and samples. In Fig. 2, the adaptation of the neural network is also depicted, showing the differences from Fig. 1.

## Evaluation

### Data

For evaluating PharmacoNER tagger, we used the SPACCC dataset, a manually annotated corpus of 1,000 clinical cases written in Spanish and annotated with mentions of chemical compounds, drugs, genes, and proteins. In particular, the set of labels consists of Normalizables (4,398 labels), No Normalizables (50 labels), Proteins (3,009 labels), and Unclear (167 labels). Note that Normalizables and No Normalizables refer to chemical entities. For instance, the word “triglicéridos” (triglycerides) is annotated as Normalizable, while “receptores de progesterona” (progesterone receptors) is labelled as Proteins. One can notice that the dataset is heavily imbalanced. The dataset has not been released yet due to an ongoing shared task (http://temu.bsc.es/pharmaconer).

### Embeddings

Eight different pre-trained word embeddings have been tested, from the Spanish Billion Word Corpus (SBWC) and data crawled from SciELO (http://www.scielo.org) and health-related categories of Wikipedia. SBWC data is general-domain, while SciELO and Wikipedia are in-domain.

‒ General domain (Universidad de Chile Spanish Word Embeddings, https://github.com/uchile-nlp/spanish-word-embeddings):

FastText embeddings from SBWC

GloVe embeddings from SBWC

- Domain-specific (PlanTL Embeddings, https://github.com/PlanTL-SANIDAD/Embeddings):

FastText Scielo

FastText medical Wikipedia

FastText Scielo + Health Wikipedia

Word2Vec Scielo

Word2Vec medical Wikipedia

Word2Vec Scielo + Health Wikipedia

### Sampling

The SPACCC dataset was split with the following proportions: 80% for training, 10% for validation, and 10% for test.

Since the dataset is imbalanced, a stratified splitting has been applied, in order to approximately maintain the same proportions of labels in the three sets.

### SPACCC POS tagger and freeling

FreeLing [16] is an open source language analysis tool suite, released under the Affero GNU General Public License of the Free Software Foundation. FreeLing was used for building the SPACCC POS Tagger (https://github.com/PlanTL-SANIDAD), which is a POS tagger trained with Spanish medical texts. SPACCC POS Tagger was successfully applied to the provided data as a pre-processing step.

### Gazetteer

The gazetteer dictionary was built with a pharmaceutical “nomenclator” maintained by the Spanish government (https://www.mscbs.gob.es/profesionales/nomenclator.do), which consists of drug names and active components of medicines. For instance, indomethacin is listed as an active ingredient, and therefore the appearances of this particular word are marked as belonging to the dictionary.

### List of affixes

The list of affixes was retrieved from a biomedical website (http://blog.nclexmastery.com/drug-stems-prefixes-roots-suffixes/) and translated into Spanish. The final format is a tab-separated file, with the type of affix (e.g., suffix, prefix, or root), the affix in English, the affix in Spanish, an example in English and the Drug class. For PharmacoNER tagger, only the columns type, and affix in Spanish are used.

### Metrics

The CONLL evaluation script is used for evaluating the results. The following overall metrics are computed: accuracy, precision, recall, F1 score. For details about the metrics, please refer to Krallinger et al.’s study [1]. For the sake of comparison, we will utilize the F1 score as the primary performance metric.

### Results with the best configuration

To train our system, we tested several configurations with various levels, which are now described:

‒ Pre-trained embeddings: Total of eight models, from general domain and in-domain.

‒ POS: Yes (using POS) and No (without POS information)

‒ Gazetteer: Yes (with gazetteer) and No (without gazetteer)

‒ Affixes: Yes (with affix information) and No (without affix information).

We trained our systems until convergence, that is until no improvement was identified in the development set for at least 10 epochs. In addition, given that the number of samples from the classes No Normalizables and Unclear are too small, we decided to discard those classes. This led our NER system to have the goal of identifying Proteins and Normalizable chemicals. First of all, to narrow down the search space, we turned off all additional features and just tested the word embeddings. Once the best embedding was found, we fixed it and tested the other configurations.

In Table 1, we present the result of our system regarding the metrics mentioned in Section 4.7. The final setting is: POS with Gazetteer turned one. One can notice that there is not a great difference between the validation and the test set in all metrics, thus meaning that no overfitting occurred.

## Conclusion

In this article we have introduced PharmacoNER tagger, a neural NER based on an existing state-of-the-art system, NeuroNER. We extended and especially adapted to the particular domain of chemical compounds in Spanish medical texts. The results in the validation set of the experiments with the SPACCC dataset have showed that the best configuration consisted of the FastText Scielo + Health Wikipedia embeddings, the POS, and the gazetteer, which have proven to be an additional source of information that can be leveraged by neural networks. In the test set, the aforementioned best configuration obtained an F1 score of 89.06. The extension to the network is domain-agnostic and could be used in other fields, but the pre-processing steps have been specifically designed for our domain.

CER involves additional challenges to the ones already present in generic NER. Our work shows that some of the sources of additional information typically used in previous CER systems based on non-neural techniques, such as affixes, can be leveraged as well by state-of-the-art neural NER systems.

We foresee that this resource will be a valuable contribution not only to semantically enhance medical texts in Spanish for pharmacological and drug-related information, but it also highlights a useful approach on how to implement medical NER taggers for languages other than English. Moreover, PharmacoNER tagger will be a useful competitive baseline system and design principle for the upcoming PharmacoNER BioNLP 2019 shared task.

## Figures and Tables

**Table 1.** Results for the best combination of embedding and features

	Accuracy		Precision		Recall		F1	
	Val	Test	Val	Test	Val	Test	Val	Test
Overall	99.59	99.66	92.37	91.35	89.7	86.89	91.01	89.06
Normalizables	-	-	94.99	93.97	88.97	85.54	91.88	89.56
Proteins	-	-	89.57	87.64	90.54	89.02	90.05	88.33

The features turned on were Part-of-Speech and Gazetteer.
